# Wet-bonding technique with ethanol may reduce protease activity in dentin-resin interface following application of universal adhesive system 

**DOI:** 10.4317/jced.60307

**Published:** 2023-05-01

**Authors:** Cláudia-Cristina-da Costa Telles, Rosanna-Tarkany Basting, Enrico-Coser Bridi, Fabiana-Mantovani-Gomes França, Flávia-Lucisano-Botelho do Amaral, Roberta-Tarkany Basting

**Affiliations:** 1 Faculdade São Leopoldo Mandic, Campinas, Brazil

## Abstract

**Background:**

Greater degradation of the hybrid layer is expected when a universal adhesive system is used, especially in the conventional application strategy. Therefore, it would important to evaluate the effect of the ethanol (ETH) and a potential matrix protease inhibitor (caffeic acid phenethyl ester/ CAPE) to maximize the ability to achieve stable dentin bond strength. The aim of this study was to evaluated the effect of ETH on a wet-bonding technique, and dentin pretreatments with different concentrations of CAPE in ethanolic solution, followed by application of a universal adhesive system (Single Bond Universal) to inhibit proteolytic activity.

**Material and Methods:**

Dentin blocks were allocated to eight experimental groups according to the strategy (total-etch our self-etch) and treatments: ETH, or dentin pretreatment with CAPE (at 0.5%, 2.5%; and 5.0%). Half of each block (each hemiblock) served as the control (without dentin pretreatments) for the same group. The bonding strategy was performed (adhesive system/ restoration with composite resin). Two slices were obtained from each hemiblock and evaluated using in situ zymography. The proteolytic activity was analyzed by quantifying the green photons of the images obtained under a fluorescence microscope in three dentin locations close to the dentin-resin interface: hybrid layer (HL), underlying dentin (UD) and deep dentin (DD).

**Results:**

Wilcoxon tests (for comparison between experimental and control groups) and Friedman and Nemenyi tests (for comparisons between interface locations) showed that there was no difference between the groups with different CAPE concentrations and the respective control groups (*p*>0.05). ETH reduced the proteolytic activity at the HL and UD (*p*<0.05).

**Conclusions:**

The wet-bonding technique with ETH proved effective in reducing the proteolytic activity. The use of CAPE in different concentrations solubilized in ethanol did not have a favorable effect on proteolytic inhibition.

** Key words:**Adhesives, Hybrid layer, Dentin, Metalloproteinases.

## Introduction

One of the main factors that affects the durability of the hybrid layer *in vivo* is the hydrolysis of the interface components, and their subsequent elution (1,2). The resulting degradation process is more intense in single-bottle, self-etching and total-etch adhesive systems due to the hydrophilic nature of their components (3). Moreover, universal adhesives are known to cause greater degradation of the hybrid layer than the two-step self-etching system (3,4).

In addition to some procedures developed to reduce the hydrolytic degradation inherent to certain adhesive systems, others have been proposed to minimize the degradation of the hybrid layer (2), including the wet-adhesion technique with ethanol (5-7). This technique can increase the longevity of the resin-dentin bond by allowing better infiltration of the resin monomer, and consequent improvement in the hybrid layer formation (8). Ethanol has a higher solvent capacity than water, and lower hydrogen bonding capacity of collagen fibrils. This allows demineralized fibrils to be chemically dehydrated, and thus create a relatively hydrophobic matrix, which reduces the hydrolysis of the interface associated with the removal of water from the substrate (5,9,10). A simplified protocol has been proposed to perform this technique, consisting of dehydration with a single, 1-minute application of ethanol (11).

Degradation of the adhesive interface is also related to the presence and activation of host-derived proteases (metalloproteinases and cysteine cathepsins) in the dentin matrix. Extracellular matrix metalloproteinases (MMPs) can degrade these proteases if collagen fibrils are incompletely infiltrated by the adhesive system (12). This will compromise the stability of the hybrid layer over time (13,14). Dentin pretreatment strategies using matrix protease inhibitors have been proposed to offset this degradation (2,6,14). Ethanol, as well as other types of alcohols, has had an inhibitory effect on MMPs (15), but has not been extensively studied. Furthermore, some additional matrix protease inhibitors can also be added to the ethanol to maximize the ability to achieve stable dentin bond strength and long-term hybrid layer stability, using a synergistic application of the inhibitor together with ethanol wet-bonding (16).

Among the matrix protease inhibitors, caffeic acid phenethyl ester (CAPE) is an important active component of bee propolis, with antioxidant, anti-inflammatory, antiviral, immunostimulating, anti-angiogenic, anti-invasive and anti-metastatic action (17,18), and stands out as an inhibitor of MMP-2 and MMP-9 (19,20). The anti-proteolytic mechanism of action of CAPE has been found to inactivate the pro-enzyme precursor of MMP proteolysis, and stimulate the activity of tissue inhibitors of metalloproteinases (TIMPs) (21). The inhibitory effect of MMPs has been shown to delay the degradation of the hybrid layer (20,22,23), but the ideal concentration of CAPE has not yet been established. Solubilized in dimethylsulfoxide (DMSO), 5% CAPE reduced nanoleakage in the hybrid layer (22), and significantly lowered the concentration of dentin MMP-2 (23) and gelatinolytic activity (20), when a conventional three-step adhesive was used, especially when applied prior to acid etching at a concentration of 0.1%. However, considering that DMSO is an aprotic and polar solvent, its evaporation from the dentin substrate can hinder that of the solvents (water and ethanol) in universal adhesive systems (24). The attraction of DMSO to hydrogen molecules (10) makes it able to absorb water. As a result, the collagen fibrils become even more impregnated by moisture, thus checking the potential effect of CAPE as an antiproteolytic agent.

The present study was developed to address the considerations that there may be greater degradation of the hybrid layer when a universal adhesive system is used, especially in the conventional application strategy (3,25), and that it is important to evaluate the effect of the ethanol wet-bonding technique and CAPE solubilized in ethanol at different concentrations. It proposes the following null hypotheses: that there is no difference between: 1) use of the ethanol wet-bonding technique and different concentrations of CAPE in ethanolic solution as a dentin pretreatment affecting proteolytic activity, when the technique is used in conventional or self-etching strategies of universal adhesive; 2) use of the ethanol wet-bonding technique and different concentrations of CAPE in an ethanolic solution as a dentin pretreatment affecting proteolytic activity at the different locations of the tooth-restoration interface.

## Material and Methods

The pretreatment agents analyzed in the study are shown in Table 1.

**Table 1:**
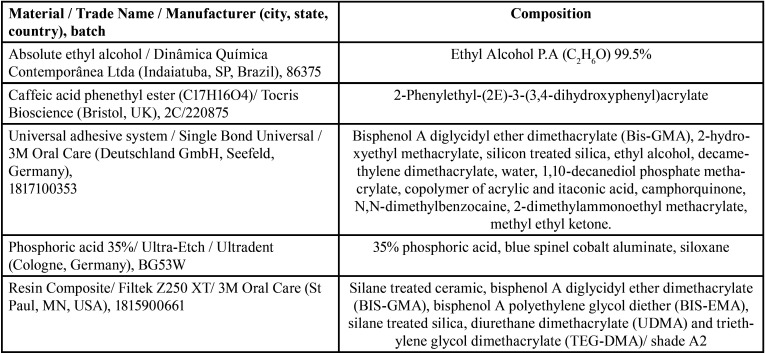
Specifications of materials used in the study.

CAPE was solubilized in ethanol. First, it was diluted in ethanol by stirring for 60 seconds to obtain a preliminary solution with a 9.29% concentration of CAPE (pH 7.98). The preliminary solution was fractionated and diluted in ethanol until concentrations of 0.5%, 2.5% and 5% were obtained. The pH of all the solutions evaluated was measured in triplicate with a microelectrode (Model 2A14, Analyser Instrumentação Analítico, São Paulo, SP, Brazil) and a pH meter (Model MPA 210, MS Tecnopon Instrumentação, Piracicaba, SP, Brazil), and yielded the following values: 8.80 for ethanol, 8.10 for CAPE at 0.5%, 8.23 for CAPE at 2.5% and 7.67 for CAPE at 5%.

-Preparation of the dentin blocks

After approval of the project by the Research Ethics Committee (CAAE 29412720.2.0000.5374), 64 sound human third molars were selected and stored frozen for a maximum period of six months. Flat surfaces of superficial dentin were obtained by removing the occlusal enamel of the teeth with a double-sided diamond disc mounted on a precision electric cutter (Isomet 1000 Precision Diamond Saw, Buehler, Lake Bluff, IL, USA), under constant cooling. Then, the surface was polished using a silicon carbide paper disc with granulation in decreasing order (400 and 600) in an electric rotary polisher (Aropol 2V, Arotec, Cotia, SP, Brazil) to obtain a standardized surface of smear layer formation. The roots of the teeth were removed 2 mm below the cementoenamel junction with a diamond disc mounted on a precision electric cutter (Isomet 1000 Precision Diamond Saw, Buehler, Lake Bluff, IL, USA), under constant cooling, and the pulp chamber was cleaned with dentin curettes.

-Dentin pretreatments 

The tooth blocks were separated into two groups according to the hybridization strategy used (conventional or self-etching). Then, each group was subdivided into four groups (n=8) according to the dentin pretreatment (ethanol, CAPE 0.5%, 2.5% or 5%). The dentin blocks were randomly allocated to each group, and their respective hemiblocks served as the control group for the same treatment. As for the treatments with the conventional adhesive strategy, 35% phosphoric acid (Ultra Etch/Ultradent) was applied for 15 seconds prior to the application of CAPE and the adhesive system, followed by removal of phosphoric acid with jets of water/air for 15 seconds, and gently drying the dentin with absorbent paper for 5 seconds, leaving it slightly moist.

Regarding the groups receiving the ethanol application (both the conventional and the self-etching strategy groups), each hemiblock of the experimental group received an application of ethanol actively on the dentin for 60 seconds with a disposable brush (Microbrush Corporation, Grafton, WI, USA), followed by gently drying the dentin with absorbent paper (Sauro *et al*., 2011). The hemiblocks of the control group received only the application of the universal adhesive system, according to the conventional or self-etching strategy, according to the manufacturer’s recommendations.

As for the groups submitted to the application of different concentrations of CAPE (both the conventional and the self-etching strategy groups), each hemiblock of the experimental group received CAPE concentrations of 0.5%, 2.5% or 5.0%, whereas the hemiblocks of the control group received no dentin pretreatment with CAPE solution. The CAPE ethanolic solution was actively applied to the dentin for 60 seconds using a disposable brush (Microbrush Corporation, Grafton, WI, USA), and then the dentin was gently dried with absorbent paper (20,23).

A layer of the universal adhesive system (Single Bond Universal/ 3M Oral Care) was actively applied for 20 seconds with a disposable brush, followed by application of an air jet for 5 seconds to volatilize and remove excess solvent. The adhesive was light-cured for 10 seconds with an LED light device (VALO, Ultradent Products, South Jordan, UT, USA) in Standard power mode, with an irradiance of 1000 mW/cm2. Then, a block with two 1-mm increments of composite resin (Filtek Z250, 3M Oral Care) was made, each increment being light-cured for 20 seconds.

-Obtaining the slices and performing the in situ zymography assays

After performing the adhesive and restorative procedures, the hemiblocks were stored with damp cotton in an incubator at 37o C. After 24 hours, two slices of approximately 500 micrometers (0.5 mm) were cut from each hemiblock. A digital caliper (Mitutoyo Sul Americana, Suzano, SP, Brazil) was used to check the thickness (26). Each slice was polished manually with 1200-grit silicon carbide sandpaper (Imperial Wetordry, 3M, Sumaré, SP, Brazil) moistened in distilled water, on both sides of each slice. Then, each slice was placed on a glass microscope slide. Gelatin (KIT DQ-gelatin, E12055; Molecular Probes, Eugene, OR, USA) was diluted in 1:8 dilution buffer (150 mM NaCl, 5 mM CaCl2, 50 mM Tris-HCl, pH 8.0), and an anti-fade agent was added (Mounting Medium, Cambridgeshire, Cambridge, UK). Subsequently, 80 µl of this mixture, containing fluorescein-conjugated gelatin, was applied to each dentin slice, and a microscopy coverslip (Microscope Cover Slips, Rochester Scientific, Rochester, NY, USA) was placed over the slices to seal them. Next, the slides were placed in a humidified chamber at 37o C for a two-hour incubation period, protected from light. Immediately after this incubation period, the slides were taken to a fluorescence microscope (Axiophoto, Zeiss, Jena, Germany) for analysis of the adhesive interface at 40x magnification, coupled to a digital camera (AxioCam HRc, Zeiss, Jena, Germany) for capturing 2D images.

-In situ zymography analysis

**Figure 1:**
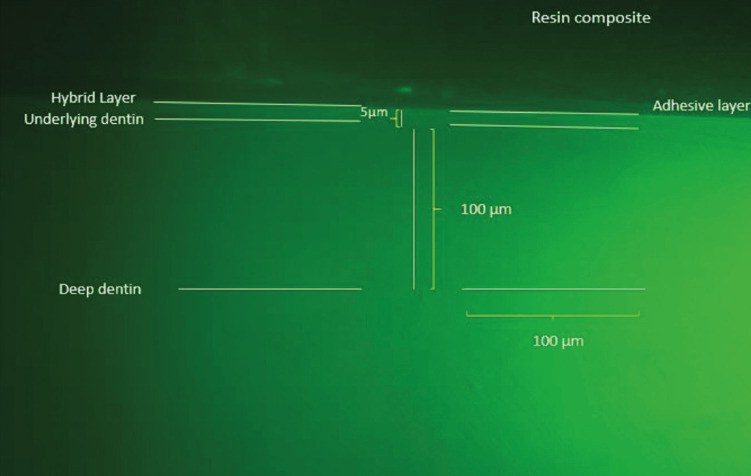
Representative adhesive dentin–resin interface analyzed in ImageJ after 24-hour incubation time with quenched fluorescein-conjugated gelatin substrate (the higher the fluorescence, the higher the enzymatic activity).

**Figure 2:**
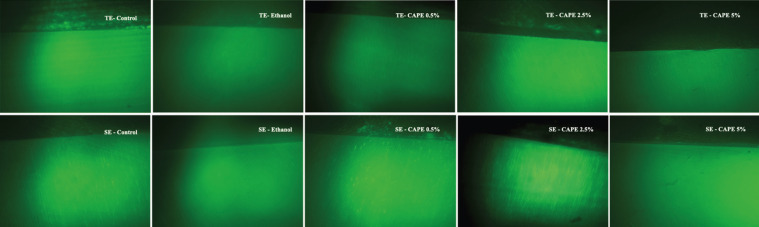
Representative dentin-resin interface images after 24-hour incubation time for all groups with quenched fluorescein-conjugated gelatin substrate, showing the different values of enzymatic activity. TE - Total-etch; SE - Self-etch.

Images were obtained along the adhesive interface of all the slices, and representative images of each group were later selected for analysis with the ImageJ program (NIH, Frederick, MD, USA). The number of fluorescent photons in red, green and blue colors were quantified in a given area using the RGB plugin (Red/Green/Blue). In in situ zymography analysis, gelatin conjugated with fluorescein emits green fluorescence when hydrolyzed and viewed under an appropriate microscope. Three locations of the adhesive interface of each slice were analyzed: hybrid layer, dentin adjacent to the hybrid layer (located at a depth of 5 μm from the hybrid layer), and deep dentin (located at 100 μm from the hybrid layer). The locations along the interface were performed randomly. Three regions each 100 μm long were selected from each of the locations for evaluation (Figs. 1,2), and the evaluation of each location was performed in triplicate, namely, one region in the center of the image, one to the left of the center, and another to the right. The format of the analyzed areas was linear, and all the areas had the same measurements (20). After obtaining the green fluorescence values of each of the three sections analyzed, an image was taken of each slice (referring to the evaluated hemiblocks), and the fluorescence values were finally submitted to statistical analysis.

-Statistical analysis

Initially, descriptive and exploratory analyses of the data were conducted, and non-parametric tests were applied. Comparisons between the experimental and control sides of the hemiblocks were performed using the paired Wilcoxon test. Comparative statistical tests were not applied between the pretreatments or between the adhesive strategies, because the pretreatments and strategies were performed on different experimental units, which may present differences in the number of proteases in their dentin matrix (27). Friedman and Nemenyi tests were used for comparisons between interface areas. All the analyses were performed in the R program (R Core Team, R Foundation for Statical Computing, Vienna, Austria, 2020), considering a significance level of 5%.

## Results

In both the hybrid layer and the adjacent dentin, the pretreatment with ethanol applied in both the conventional and self-etching strategies presented a lower number of photons in the experimental hemiblock than in the control hemiblock (*p*<0.05) (Table 2). Fewer fluorescent photons were observed in the hybrid layer when ethanol was used in the conventional strategy, and fewer also in the hybrid layer and adjacent dentin when the self-etching strategy was used (*p*<0.05).

**Table 2:**
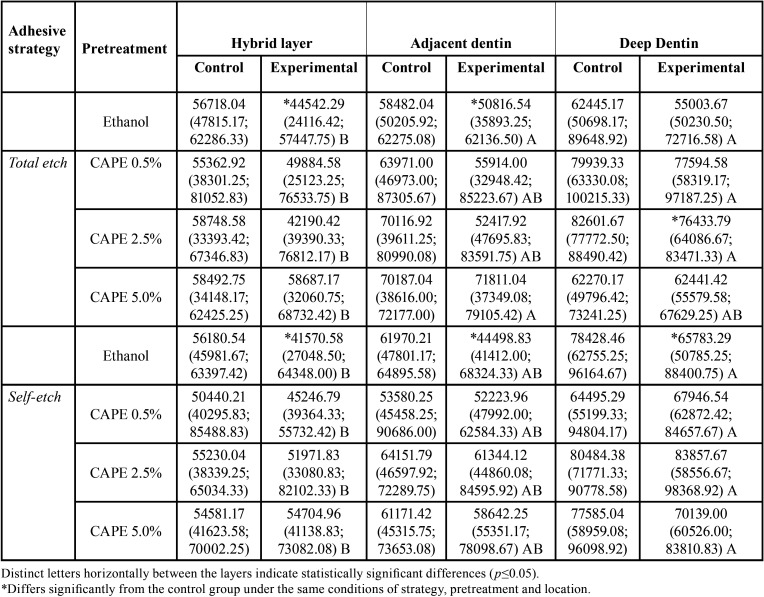
Number of fluorescent photons identified by green staining according to adhesive strategy, dentin pretreatment and location of the adhesive interface.

All the groups using CAPE had lower values of fluorescent photons in the hybrid layer compared to deep dentin (*p*<0.05). In deep dentin, the 2.5% CAPE pretreatment in the conventional strategy showed a lower number of photons on the experimental side of the hemiblock than on the control side (*p*=0.0148). The hybrid layer presented a lower number of fluorescent photons than the other layers (*p*<0.05).

## Discussion

The present study showed that the wet-adhesion technique with ethanol promoted lower dentinal proteolytic activity, regardless of the adhesion strategy, thus calling for the rejection of the first null hypothesis. Ethanol has been described as an agent that reduces the fibrillar diameter of collagen, and increases the interfibrillar space in the hybrid layer, thus facilitating infiltration of resin monomers between the collagen fibers, and preventing them from being exposed (13,28,29). This aspect becomes especially important when the conventional adhesive strategy is used, since the depth of dentin demineralization by phosphoric acid is greater than the penetration capacity of the resin monomers (8,28,29).

The association between universal adhesives and the wet-bonding technique with ethanol provided an increase in bond strength to dentin, when used in both the conventional and self-etching strategies, even after simulating thermomechanical aging (8). It has been suggested that ethanol can interact with the smear layer, thus modifying the organic matrix to facilitate the penetration of adhesive monomers, and ultimately lead to a more effective encapsulation of collagen fibrils. In addition, the action of a universal adhesive in the self-etching mode on the dentin substrate can be enhanced by using ethanol as a vehicle to favor the bonding of 10-MDP to calcium ions, considering that hydroxyapatite crystals are maintained in the dentin substrate (8).

Ethanol has also been shown to reduce the permeability of the hybrid layer by reducing water sorption and hydrolysis of the adhesive and hybrid layer, thus making the hybrid layer more sTable (7,30,31). This outcome may be related to the ethanol-related increase in interfibrillar spaces. A greater number of these spaces allow improved impregnation, and increase the number of resin monomers responsible for protecting collagen fibrils from degradation, and improving the mechanical properties of the adhesive interface (5,32). The present study observed not only the effect of ethanol in reducing hydrolytic degradation, but also the reduction in protease activity in the dentin matrix, corroborating the studies by Tezvergil-Mutluaya *et al*. (15). A possible explanation for this inhibition is that alcohols usually inhibit MMPs, since they are able to establish a covalent bond between the zinc catalytic site of MMPs and the hydroxyl oxygen atom of alcohols. This is how MMPs are inactivated, and the durability of the resin-dentin bond is thus improved (3).

The inhibition of proteases by using ethanol was significant in the hybrid layer and in the adjacent dentin when the conventional adhesion strategy was used, thus rejecting the second null hypothesis of the present study. It is known that the gelatinolytic activity in the lower part of the hybrid layer (adjacent dentin) is intense (27), and can contribute toward the relatively rapid loss of bond strength in conventional adhesive systems (33-35). The location of the gelatinolytic activity correlates well with the layer of demineralized collagen not infiltrated by the adhesive in the conventional technique on the underside of the hybrid layer (36).

In the self-etching strategy, the ethanol used for dentin pretreatment was able to inhibit enzymatic activity in all layers/locations of the hybrid layer. This can be explained by the non-use of phosphoric acid, with simultaneous demineralization of dentin and involvement of collagen fibrils, when using the universal adhesive system. The activation of MMPs by dentinal adhesives is known to increase linearly with the decrease in their pH (37). Ethanol used for dentin pretreatment allows a significant amount of water to evaporate in these spaces, and another part of the water to evaporate from the action of the solvent present in the universal adhesive system, which also has alcohol in its composition (8,29,38).

The use of CAPE at different concentrations solubilized in ethanol did not have a favorable effect on protease inhibition, compared to the control group. Although CAPE has been reported to promote selective inhibition of MMP-2 and MMP-9 expression (19,38), this effect was not observed in the present study. The CAPE concentrations (0.5%, 2.5% and 5%) in ethanol were higher than those of the studies by Pedrosa *et al*. (23) and Pedrosa *et al*. (19), who used 0.05% and 0.1% CAPE solubilized in DMSO. This may suggest that the CAPE concentrations in ethanol used herein are not effective for immediate enzyme inhibition, and also affected the protease inhibition of ethanol. It is recognized that the hydrolysis of esters (such as the phenethyl ester of caffeic acid) leads to acid hydrolysis, in which the acidic medium (H+) catalyzes the reaction, producing acid and alcohol until an equilibrium is reached between the reactions (39). The great hydrophilicity of the universal adhesive used, and the increase in the temperature of the medium from the use of photoactivation, can be considered as catalytic agents triggering the hydrolysis of CAPE.

Although the inhibitory action of CAPE at different concentrations solubilized in ethanol was not proven in the present research, long-term studies should be carried out to better understand the influence of CAPE in protease inactivation in the hybrid layer, when used with a universal adhesive system. It’s also noteworthy that the methodology used to evaluate the activation of different dentinal proteases was not specific for any specific type of metalloproteinases or cysteine-cathepsins, and that in situ zymography with fluorescence-labeled gelatin is adequate and accurate enough to investigate the involvement of proteases in the degradation of the hybrid layer (40). It should also be pointed out that the clinical use of the wet-adhesion technique with ethanol is viable, both in terms of its application and its cost effectiveness, and that further studies are needed to determine the applicability of using CAPE in different solutions and concentrations with other analytical methodologies.

Dentin pretreatment with ethanol using the wet-adhesion technique proved effective in reducing the proteolytic activity of the hybrid layer and adjacent dentin, regardless of the adhesion strategy used. The use of CAPE in different concentrations solubilized in ethanol did not have a favorable effect on inhibiting enzymatic activity at the tooth-restoration interface.
